# Optimization of Electrochemical Treatment Process Conditions for Distillery Effluent Using Response Surface Methodology

**DOI:** 10.1155/2015/581463

**Published:** 2015-09-29

**Authors:** P. Arulmathi, G. Elangovan, A. Farjana Begum

**Affiliations:** ^1^Department of Civil Engineering, University College of Engineering (Anna University), Dindigul, Tamil Nadu 624622, India; ^2^Department of Civil Engineering, University College of Engineering (Anna University), Pattukkottai, Tamil Nadu 614701, India; ^3^Tiruchirappalli District Cooperative Milk Producer's Union Ltd., Trichy, Tamil Nadu 620023, India

## Abstract

Distillery industry is recognized as one of the most polluting industries in India with a large amount of annual effluent production. In this present study, the optimization of electrochemical treatment process variables was reported to treat the color and COD of distillery spent wash using Ti/Pt as an anode in a batch mode. Process variables such as pH, current density, electrolysis time, and electrolyte dose were selected as operation variables and chemical oxygen demand (COD) and color removal efficiency were considered as response variable for optimization using response surface methodology. Indirect electrochemical-oxidation process variables were optimized using Box-Behnken response surface design (BBD). The results showed that electrochemical treatment process effectively removed the COD (89.5%) and color (95.1%) of the distillery industry spent wash under the optimum conditions: pH of 4.12, current density of 25.02 mA/cm^2^, electrolysis time of 103.27 min, and electrolyte (NaCl) concentration of 1.67 g/L, respectively.

## 1. Introduction

Distilleries are an important industrial sector in India where ethyl alcohol is produced from the cane sugar molasses by fermentation process. At the end of fermentation, broth containing 6–8% alcohol by volume is distilled to recover alcohol. Alcohol is separated by distillation and the residual liquor is discharged as effluent called “spent wash.” Distillery spent wash contains high content of organic matter and it is generally dark brown liquid with an unbearable odor. When it is discharged without proper treatment it will lead to change in color and depletion of dissolved oxygen concentration of the receiving water stream [1].

Different treatment techniques are used to treat the distillery spent wash before its disposal on land or water environment. Several physical methods have been employed for the removal of organic matters from the effluent, but they have many disadvantages such as low removal efficiency and being expensive. Of the various techniques for the treatment of distillery spent wash, biomethanation has gained wide acceptability due to methane recovery in the anaerobic step of the treatment. It is reported that biological treatment results in 60–85% of the BOD reduction, but still substantial amount of recalcitrant organic pollutants is left behind in the effluent discharge [2]. Electrochemical treatment method (EC) is an attractive, alternative treatment process and has several advantages over conventional treatment methods such as easy automation, maximum removal efficiency, shorter treatment time, low sludge production, and reasonable operating cost.

In this electrochemical treatment process, electrooxidation methods were widely investigated. Electrooxidation is a process in which the pollutants are destroyed or converted into simpler forms like carbon dioxide and water. This oxidation process can be either direct or indirect oxidation. In the indirect oxidation process, strong oxidants like hypochlorite, chlorine, hydrogen peroxide, and hydroxyl ions are electrochemically generated at the anode [3]. All the oxidants are produced in situ and are utilized immediately. The mechanism of indirect electrochemical oxidation has suggested that water is electrolyzed by anodic catalysis to produce hydroxyl radicals, being shown in

(1) MOx+OH–⟶MOxOH∙+e−

(2) MOxOH∙+D⟶xCO2+xe−+zH+

(3) MOxOH∙+D⟶12O2+e−+MOx

Meanwhile, in the presence of chloride ion, another strong oxidant of hypochlorous acid may be produced on the anode during electrolysis and will react with organic matters:

(4) H2O+Cl−⟶HClO+H++2e−

(5) HClO+R⟶product+Cl−

where *M* is the anode site and *D* is the color causing organic molecule. These equations indicate that oxidation intermediates are formed which is then degraded, resulting in destruction of the organic pollutant and color treatment. Different types of electrodes for indirect electrochemical oxidation have been investigated by various researchers for the treatment of various industrial wastewaters. An electrochemical-oxidation process is influenced strongly by the nature of the anode material [4]. Titanium coated with boron doped diamond (Ti/BDD) was tested for domestic wastewater. Lead oxide coated on expanded titanium mesh (Ti/PbO_2_) has been widely used in EC treatment due to its easy preparation, chemical stability, and high potentials for oxygen evolution [4]. Electrochemical-oxidation methods are likely to gain a better public acceptance than the alternative methods for environmental cleanup. Optimization of EC treatment process conditions will pave the way for minimizing the operating cost with maximum treatment efficiency. Statistic-based experimental designs have proved to be more efficient than classical one-at-a-time method, which is tedious and time-consuming, do not screen multivariables, and do not inspect the complex interactions among different factors. Besides, statistical approaches provide a systematic and efficient plan for experimentation to achieve certain goals. These methods will reduce the number of experiments; in addition to that it also considers the interactions of many factors which may affect the removal efficiency during treatment process. Response surface methodology (RSM) is a statistical technique, widely used to optimize the operational variables for experiment designing, modeling, and so forth. There are various advantages in using statistical methodologies, in terms of rapid and reliable selection of process conditions, examining the effective factors, and minimizing the error in determining the effect of parameters [5]. In this study process variables of indirect electrochemical-oxidation process such as pH, current density, electrolysis time, and electrolyte dose were optimized to remove chemical oxygen demand (COD) and color from distillery industry spent wash using Box-Behnken response surface design (BBD).

## 2. Materials and Methods

### 2.1. Materials

Distillery spent wash was collected from the industry near Theni District, Tamil Nadu, India. The characteristics of distillery spent wash were determined using APHA standard methods [6]. It was shown in Table 1.

### 2.2. Experimental Setup

**Figure 1:**
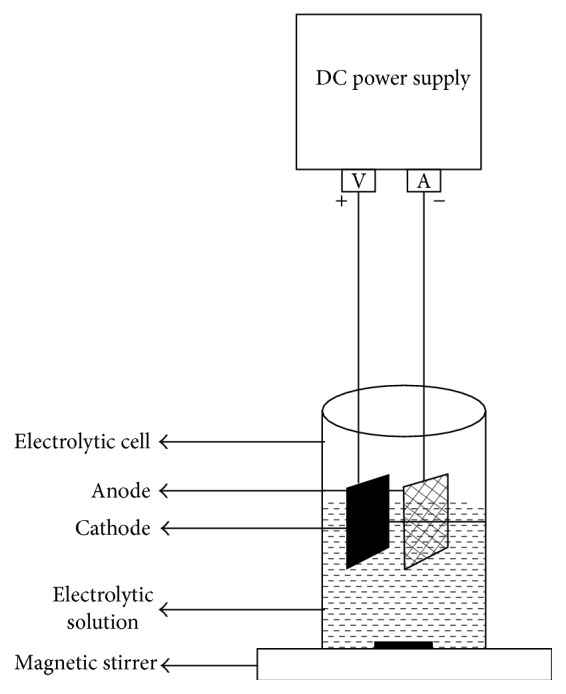
Experimental setup for electrochemical method.

The experimental setup used in this study is shown in Figure 1, which mainly consisted of a beaker of 1200 mL as a reactor to hold a sample of 1000 mL. The electrodes employed were mesh type Ti/Pt (10 mm × 10 mm) anode and Ti (10 mm × 10 mm) as cathode. The desired current density was maintained constant by means of a precision digital direct current power supply (0–30 V, 0-1 A). All experiments were conducted in batch mode of operation and pH of the spent wash was adjusted using 0.1 N HCl or NaOH. In each experimental run, a sample was rigorously stirred to avoid concentration gradients. The electrodes were washed with HCl solution (5% W/V) before each run. Following each run, the electrodes were washed, dried, and used again. At the end of each batch experiment, the sample was transferred and allowed to settle down. After a settling time of 20 min, the supernatant sample was collected to perform the analysis of COD and color.

The efficiency of the EC process was determined in terms of the COD using the following equation:

(6) $$\begin{array}{c} R=\frac{\left(Y_{0}-Y_{1}\right)}{Y_{0}}\times 100, \end{array}$$

where *R* is the COD removal efficiency (%), *Y*_0_ is initial COD of the sample, and *Y*_1_ is final COD of the sample in mg O_2_/L.

### 2.3. Experimental Design

In this present study, the experimental design and statistical analysis were performed according to the RSM using Design-Expert software (trial version 7, Stat-Ease). Box-Behnken response surface experimental design (BBD) was used to optimize EC process variables such as pH, current density, electrolysis time, and electrolyte dose on the treatment of distillery industry spent wash using electrochemical method (EC). Single factor experimental analysis was used to select the range of process variables. The factors were examined at three different levels (low, basal, and high) coded (−1, 0, and +1). The actual factor levels and coded values of independent variables used for treatment of spent wash are given in Table 2.

Experiments were established based on a BBD which consists of 29 experiments with five centre points. The total number of experiments was calculated as follows:

(7) $$\begin{array}{c} N=2K\left(\;K-1\;\right)+C_{0}, \end{array}$$

where *K* is number of factors and number of central points is denoted by *C*_0_.

The relationship between the operating variables and the response is described in the following empirical quadratic polynomial equation:

(8) $$\begin{array}{c} Y=\beta_{0}+\overset{k}{\underset{i=1}{\sum }}\beta_{i}X_{i}+\overset{k}{\underset{i=1}{\sum }}\beta_{ii}X_{i}^{2}+\overset{k-1}{\underset{i=1}{\sum }}\;\overset{k}{\underset{j=2}{\sum }}\beta_{ij}X_{i}X_{j}, \end{array}$$

where *Y* is the response variable, *β*_0_ is the constant, *β*_*i*_ is the linear coefficient, *β*_*ii*_ is the quadratic coefficient, and *β*_*ij*_ is the interaction coefficients; *X*_*i*_ and *X*_*j*_ represent the coded independent variables. *k* is the number of parameters; and *e*_*i*_ is the error. The regression coefficients of linear, quadratic, and interaction were determined by the software, and the significance of all terms was assessed statistically using *F* value at a probability (*P*) of 0.001, 0.01, or 0.05. They were further used to make statistical calculations to generate contour maps with the help of the regression model. The contour plots were generated by keeping three variables constant at the center point and varying the other two variables within the experimental range [7].

## 3. Result and Discussion

BBD design matrix of the independent variables in coded units (experimental design) along with experimental values of response is given in Table 3.

The results obtained from BBD experiments were analyzed by multiple regression analysis.  Table 4 displays the regression coefficient and the probability (*P*) values. A smaller *P* value denotes greater significance of the corresponding coefficient. The effects of four independent variables including pH, electrolysis time (min), current density (mA/cm^2^), and electrolyte concentration (g/L) on removal efficiency are shown in Table 4.

The effects of pH (*X*_1_), electrolysis time (*X*_2_), and electrolyte concentration (*X*_4_) on both COD and color removal efficiency were statistically significant (*P* < 0.05), but the effect of current density on color removal efficiency is not statistically significant. Among the interaction combinations, combinations *X*_1_ *X*_2_, *X*_2_ *X*_3_, and *X*_2_ *X*_4_ were found to have significant effect (*P* < 0.05) on COD removal, whereas *X*_1_ *X*_3_, *X*_1_ *X*_4_, and *X*_2_ *X*_4_ have significant effect (*P* < 0.05) on color removal in distillery spent wash. In addition, the three quadratic terms *X*_1_^2^, *X*_2_^2^, and *X*_3_^2^ have significant effects (*P* < 0.05) on both COD and color removal in spent wash, whereas *X*_4_^2^ have significant effect on COD removal alone and there are no effects on color removal. After using the designed experimental data and eliminating some terms, two empirical models were developed by considering only the significant terms (*P* < 0.05). The final models obtained in terms of coded factors are given in

(9) % of  COD  removal=86.19+6.99·X1+8.94·X2−5.08·X3+9.21·X4+6.87·X1X2+6.09·X2X3−7.83·X2X4−6.27·X12−16.14·X22−4.64·X32−9.71·X42,% color  removal=91.22+10.98·X1+12.47·X2+9.52·X4−2.75·X1X2−4.77·X1X3−4.5·X1X4−11.95·X2X4−10.82·X12−20.37·X22−4.94·X32.

The regression model was further evaluated using analysis of variance (ANOVA), and the results are shown in Table 5. The low probability value demonstrates that the model is highly significant. The goodness of fit of the model was further checked using the determination coefficient (*R* ^2^); the values are *R* ^2^ = 95%. Furthermore, the value of *R* ^2^ also indicates that the model can explain 95% of the total variation for the COD removal and color removal efficiency to the operating parameters. To visualize the relation between the operating variables and removal efficiency, linear plots and contour maps were generated and presented using Design-Expert (version 7.0) based on the regression model.

**Figure 2:**
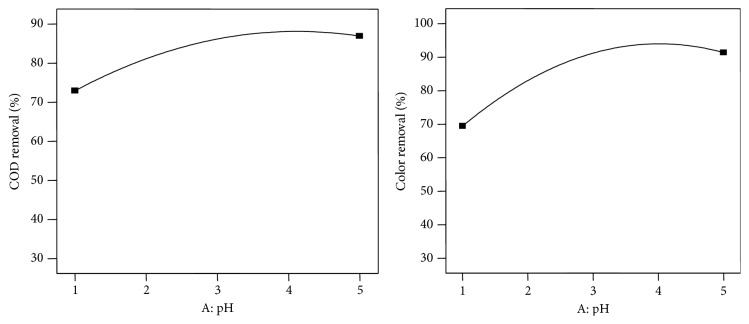
Effect of pH on COD and color removal.

The graphical representations of the linear effect of the variables were developed using Design-Expert (version 7.0) software. To examine pH effect on the treatment efficiency, pH ranges are varied from 1 to 5 (Figure 2). The results showed that the percentage of COD and color removals are increased with increasing pH up to 4.5. It indicates that acidic condition is more favorable for the treatment of distillery spent wash. In acidic conditions, hypochlorous acid was the major compound in the solution. Therefore higher rate of decolorization and degradation in acidic condition may be due to higher oxidation potential of hypochlorous acid. Above pH 5, the rate of reaction was lowest. This result can be attributed to undesirable side reactions such as oxidation of free chlorine to chlorate and perchlorate, reactions involving loss of hypochlorite.

**Figure 3:**
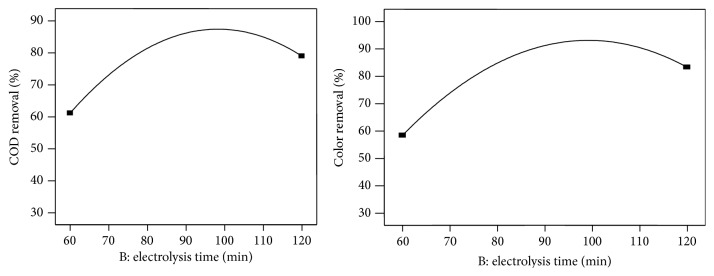
Effect of electrolysis time on COD and color removal.

Electrolysis time is an important parameter for controlling the reaction rate in electrochemical treatment process. The linear effect of electrolysis time on COD and color removal efficiency was studied during electrochemical treatment process by varying the electrolysis time from 60 to 120 min, and the results are shown in Figure 3. From the observation, it is found that the percentage of COD and color removal was increased with increasing of electrolysis time up to 100 min [8].

**Figure 4:**
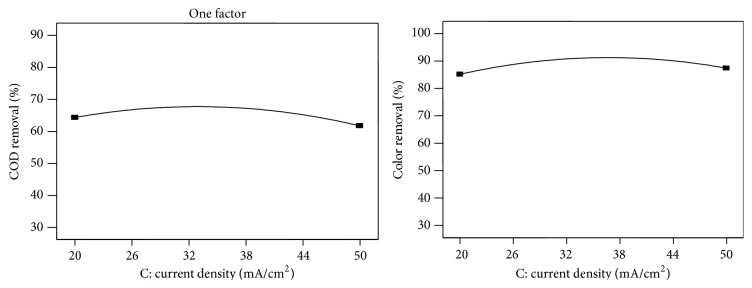
Effect of current density on COD and color removal.

The linear effect of current density on COD and color removal efficiency was studied during electrochemical treatment process by varying the current density from 20 to 50 mA/cm^−2^, being shown in Figure 4. From the results, it is found that COD and color removal increase with increasing of current density. This is attributed to the higher formation of ion species, which has the strong affinity towards the organic matters present in the wastewater to be treated; thus removal efficiencies were increased [9].

**Figure 5:**
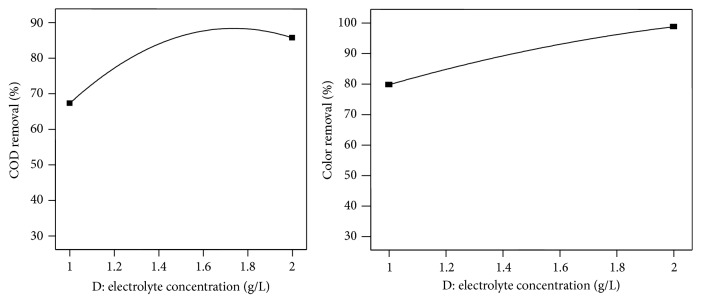
Effect of electrolyte concentration on COD and color removal.

The effect of another important process parameter (electrolyte concentration) on COD and color removal efficiency was studied during an electrochemical-oxidation treatment process by varying the concentration of NaCl from 1 to 2 g/L (Figure 5). From the graph, it is noticed that the removal efficiency of COD and color increases with increasing electrolyte concentration. The concentration of electrolyte affected the conductivity. The conductivity was increased by the addition of sodium chloride. Chloride ions could significantly reduce the adverse effects of other anions, such as HCO^3−^ and SO_4_^2−^, that could form an insulating layer on the surface of the electrodes and increase the ohmic resistance of the electrochemical cell. The increase in the removal efficiency of COD and color may be attributed to a change in the ionic strength due to the increasing conductivity of aqueous medium [10].

**Figure 6:**
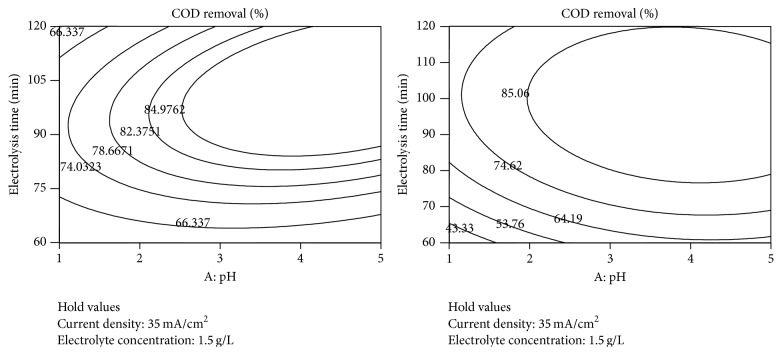
Contour plots between pH and electrolysis time for the responses.

**Figure 7:**
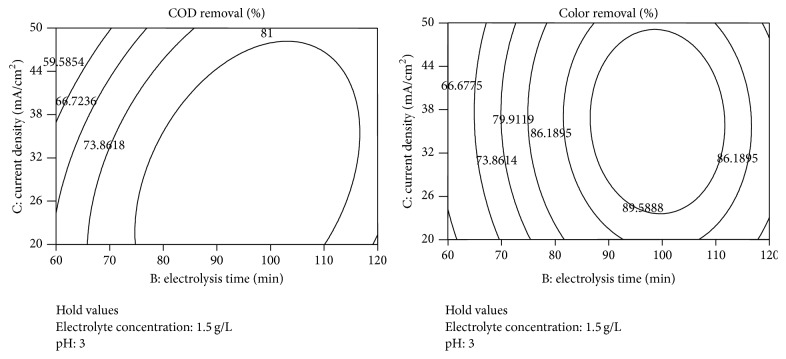
Contour plots between electrolysis time and current density for the responses.

**Figure 8:**
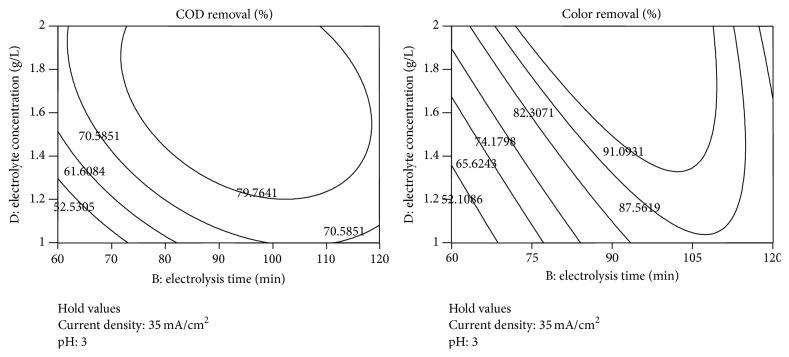
Contour plots between electrolysis time and electrolyte concentration for the responses.

The graphical representations of the interaction effect of the variables called the contour plots were developed using Design-Expert (version 7.0) software. Interaction between any two test variables was studied during electrochemical treatment process, keeping the other two variables constant at their middle level [11]. Circular or elliptical nature of the contours shows the interactions between the two independent variables being significant or not. Contour plots of the interactive effect of pH, electrolysis time (min), current density (mA/cm^2^), electrolyte concentration (g/L) on removal efficiency are given in Figures 6–8 showing the response.

The interaction effects of pH and electrolysis time (min) on COD and color reduction are shown in Figure 6, while the other variables (current density and electrolyte concentration) were fixed at central level (35 mA/cm^2^ and 1.5 g/L, resp.). The elliptical shape of the contour plots between pH and electrolysis time (min) indicates that there is a significant interaction effect between these variables during electrochemical treatment process. Simultaneously increasing the two variables caused a linear increase in the COD and color removal efficiency. However, a decrease in the removal efficiency was observed with further increases in the two variables. The maximum values of COD and color removal were found to be 85% and 83% respectively, when pH and electrolysis time were found to be approximately 4 and 97 min, respectively.

The contour map in Figure 7 shows the interaction between electrolysis time (min) and current density (mA/cm^2^) on COD and color removal in spent wash, when the other two variables were fixed at centre level. According to Figure 7, the contours around the stationary point were circular and get elongated more along the current density axis. So a lower value of current density and electrolysis time (min) increased the percentage removal of COD and color in distillery spent wash. The results showed that the color removal efficiency increases with the increasing of current density [12].

The contour map based on two variables, electrolysis time (min) and electrolyte concentration (g/L), is shown in Figure 8, whereas the other two variables were kept at a centre level. It can be seen that the two variables had a positive impact on both COD and color removal efficiency. At the lowest level of electrolysis time and electrolyte concentration, the removal efficiency of spent wash was found to be increase slightly at first and then decrease with continuous increasing of electrolysis time and electrolyte concentration, respectively. The maximum COD removal of spent wash was found to be 80% and Color removal of spent wash was found to be 91%. The addition of NaCl into spent wash increases conductance and electric current of electrochemical process. Increasing solution conductivity resulted in the reduction of cell voltage that caused a decrease in electrical energy consumption. Also, solution resistance and the potential in solution decrease with the enhancement of electrolyte concentration. In this study, the removal of reactive organic compounds increased with the increasing of NaCl concentration. The complete removal obtained above 1.2 g/L NaCl and 90 min electrolysis time. The results of the present investigation have shown that the degradation occurred very efficiently in the presence of NaCl as a conductive electrolyte. It could be concluded that the degradation was faster when NaCl was used as electrolyte in the study. In most cases, high concentrations of supporting NaCl electrolyte are required for satisfactory results leading to high concentrations of hypochlorite anions and free chlorine. However, the use of NaCl involves the possibility of the formation of organochloride compounds, mainly due to the presence of OCl^−^ as a possible side reaction [12].

In order to find out the optimum operating conditions to treat distillery industry spent wash using electrochemical treatment method, simultaneous optimization of the multiple responses is carried out. Here, process parameters (A–D) were selected as within range and responses (*Y*_1_ and *Y*_2_) fixed as a maximize. Optimal operating conditions to obtain the maximum removal of COD and color are determined as follows: pH of 4.12, electrolysis time of 103.27 min, current density of 25.02 mA/cm^2^, and electrolyte dose of 1.67 g/L, respectively. Under these optimum conditions, the COD removal efficiency and color removal efficiency were found to be 89.5% and 95.1%, respectively, which are validated by conducting additional experiments under the optimal conditions. A mean value of 88.6% for COD removal and 94.65% of color removal are obtained from the experiment which are in close agreement with the predicted values obtained from numerical optimization technique. The good correlation between these observed results and predicted values indicates the reliability of BBD incorporate desirability function method and it could be effectively used to optimize the EC process parameters.

## 4. Conclusion

The electrochemical treatment of distillery spent wash was investigated with Ti/Pt electrodes. The effects of EC process parameters such as pH, current density, electrolyte concentration, and electrolysis time on COD and color removal were found batchwise. The best operational conditions for COD and color removal were attained in pH of 4.12, electrolysis time of 103.27 min, current density of 25.02 mA/cm^2^, and NaCl dose of 1.67 g/L, respectively. During the electrochemical treatment, rapid decoloration and increased COD removal were achieved throughout the study indicating that the organic compounds and other pollutants were completely degraded into smaller, colorless organic and inorganic molecules. For the use of electrochemical treatment in industrial applications, treatment process was experimentally designed and optimized through response surface methodology, which is a promising and cost economical approach to remove organic pollutants present in distillery industry spent wash.

## Figures and Tables

**Table 1:** Characteristics of distillery industry spent wash.

Serial number	Characteristics of effluent	Value
1	pH	4.5–5
2	Solubility in water (%)	100
3	Colour	Dark brown
4	COD (mg O_2_/L)	100000–130000
5	BOD (mg O_2_/L)	55000–65000
6	Odour	Burnt gelatin/glucose
7	Total solids	130000–160000
8	Total dissolved solids	35000–45000
9	Total volatile solids	60000–75000

**Table 2:** Ranges of independent variables and their levels.

Independent variables	Coded symbol	Coded values and actual factor levels		
		−1	0	+1
pH	*X*_1_	1	3	5
Electrolysis time (min)	*X*_2_	60	90	120
Current density (mA/cm^2^)	*X*_3_	20	35	50
Electrolyte concentration (g/L)	*X*_4_	1	1.5	2

**Table 3:** Experimental design, observed yields in BBD experiments in terms of percentage removal of COD and color.

Run	pH	Electrolysis time (min)	Current density (mA/cm^2^)	Electrolyte concentration (g/L)	COD removal (%)	Color removal (%)
1	5	90	20	1.5	84.54	89.22
2	3	90	35	1.5	85.56	92.18
3	5	120	35	1.5	83.86	83.32
4	5	90	35	1.0	76.52	86.27
5	1	90	20	1.5	76.22	60.47
6	5	90	35	2.0	87.22	92.33
7	3	60	20	1.5	68.99	45.26
8	3	60	35	1.0	32.15	37.24
9	3	90	35	1.5	86.43	91.18
10	1	90	35	1.0	48.52	52.33
11	3	90	50	1.0	57.12	82.31
12	3	60	50	1.5	45.96	50.25
13	3	60	20	2.0	74.49	76.37
14	3	90	50	2.0	84.45	94.42
15	3	120	50	1.5	74.37	80.43
16	1	60	35	1.5	59.91	35.19
17	3	90	35	1.5	86.43	91.16
18	3	120	35	2.0	69.38	76.42
19	3	90	35	1.5	86.48	91.23
20	3	90	35	1.5	86.75	91.16
21	1	120	35	1.5	61.18	67.09
22	3	60	35	2.0	61.96	82.89
23	3	120	20	1.5	74.43	80.36
24	3	90	20	1.0	65.76	71.32
25	1	90	35	2.0	71.36	76.44
26	5	90	50	1.5	73.24	80.21
27	5	60	35	1.5	55.12	62.42
28	3	120	35	1.0	69.49	76.91
29	1	90	50	1.5	59.45	70.54

**Table 4:** Regression coefficients obtained by the response surface model.

Term constant	COD reduction (%)		Color reduction (%)	
	Regression coefficient	*P* value	Regression coefficient	*P* value
Intercept	86.19	<0.0001	91.01	<0.0001
*X*_1_	6.99	<0.0001	10.97	<0.0001
*X*_2_	8.94	<0.0001	12.47	<0.0001
*X*_3_	−5.08	0.0014	1.12	0.3784
*X*_4_	9.21	<0.0001	9.52	<0.0001
*X*_1_ *X*_1_	−6.27	0.0027	−10.87	<0.0001
*X*_2_ *X*_2_	−16.14	<0.0001	−20.37	<0.0001
*X*_3_ *X*_3_	−4.64	0.0167	−4.94	0.0092
*X*_4_ *X*_4_	−9.71	<0.0001	−1.93	0.2586
*X*_1_ *X*_2_	6.87	0.0070	−2.75	0.2083
*X*_1_ *X*_3_	1.37	0.5894	−4.77	0.0382
*X*_1_ *X*_4_	−3.04	0.1843	−4.51	0.0482
*X*_2_ *X*_3_	−6.09	0.0110	−0.81	0.6895
*X*_2_ *X*_4_	−7.83	0.0021	−11.95	<0.0001
*X*_3_ *X*_4_	1.86	0.4384	−3.66	0.0559

**Table 5:** ANOVA for response.

Source	COD reduction (%)		Color reduction (%)	
	*F* value	*P* value	*F* value	*P* value
Model	20.24	<0.0001	32.33	<0.0001
*A*	31.01	<0.0001	83.14	<0.0001
*B*	52.20	<0.0001	110.41	<0.0001
*C*	15.20	0.0014	0.83	0.3784
*D*	51.24	<0.0001	59.51	<0.0001
*AB*	9.98	0.0070	1.74	0.2083
*AC*	0.40	0.5394	5.23	0.0382
*AD*	1.95	0.1843	4.68	0.0482
*BC*	8.57	0.0110	0.17	0.6895
*BD*	14.17	0.0021	35.91	<0.0001
*CD*	0.64	0.4384	2.68	0.0559
*A* ^2^	13.26	0.0027	42.92	<0.0001
*B* ^2^	87.68	<0.0001	151.77	<0.0001
*C* ^2^	7.38	0.0167	9.10	0.0092
*D* ^2^	32.36	<0.0001	1.39	0.2586
Lack of fit	129.95	0.0001	121.42	0.0002
*R* ^2^	0.9529		0.9754	
Adj-*R* ^2^	0.9058		0.9508	
